# Enhanced Impulsive Action Selection in Middle-Aged Adults—Insights From an Oculomotor Simon Task

**DOI:** 10.3389/fnagi.2016.00251

**Published:** 2016-10-25

**Authors:** Joan Duprez, Jean-François Houvenaghel, Soizic Argaud, Florian Naudet, Thibaut Dondaine, Manon Auffret, Gabriel Robert, Dominique Drapier, Marc Vérin, Paul Sauleau

**Affiliations:** ^1^“Behavior and Basal Ganglia” Research Unit (EA 4712), University of Rennes 1Rennes, France; ^2^Neurology Department, Rennes University HospitalRennes, France; ^3^Neuroscience of Emotion and Affective Dynamics Laboratory, Swiss Center for Affective SciencesGeneva, Switzerland; ^4^Adult Psychiatry Department, Rennes University HospitalRennes, France; ^5^Clinical Investigation Center (INSERM 0203), Department of Pharmacology, Rennes University HospitalRennes, France; ^6^Neurophysiology Department, Rennes University HospitalRennes, France

**Keywords:** aging, cognitive action control, Simon task, activation-suppression, distributional analyses, selective inhibition

## Abstract

Several studies have investigated the age-related impact in cognitive action control. However, to our knowledge, none of the studies have focused on the effect of moderate age on the strength of automatic activation according to the activation-suppression model. We therefore investigated the effect of moderate age on cognitive action control using an oculomotor version of the Simon task and distributional analyses. A group of middle-aged (*n* = 39; 57 ± 9 years) healthy adults were compared to a group of young healthy participants (*n* = 43; 24 ± 3 years). We first analyzed the overall impact of age on the congruence effect and then used conditional accuracy functions (CAFs) and delta plots to assess the strength of automatic activation and selective inhibition, respectively. Compared to young participants, middle-aged participants showed a greater congruence effect as well as higher rates of fast errors in conflict situations indicating an enhanced impulsive action selection. Furthermore, the overall downward slope of the congruence effect’s evolution was significantly steeper in older participants and the last slope tended to be significantly steeper. This may indicate that the middle-aged participants exerted a stronger selective inhibition. Our results suggest that middle-aged adults are more prone to impulsive action selection than young adults. Recent theories postulate that older adults might implement compensatory mechanisms to supply cognitive difficulties. This is in line with our results suggesting a potential greater selective inhibition. Overall, this study proposes that moderate aging impacts both processes of impulsive response selection and suppression underlying cognitive action control.

## Introduction

As they get older, adults experience increasing difficulties with cognitive functions such as working memory and attention. This has given rise to several interpretations and theories (Lustig and Jantz, 2015). One of the functions affected by age in healthy adults is the *cognitive action control*, namely, the cognitive process that tailors our behaviors to our goals. For instance, in case of conflict between two or more action activations, it enables us to inhibit unwanted response tendencies and select the most relevant action for our purpose, depending on the context.

Conflict tasks such as the Eriksen flanker task, the Stroop task or the Simon task have been extensively used to investigate this process (Stroop, 1935; Simon, 1969; Wallace, 1971; Eriksen and Eriksen, 1974; Hedge and Marsh, 1975; Burle et al., 2002; Egner et al., 2007). In the Simon task, stimuli have both a relevant (color) and an irrelevant (location) dimension. Participants have to press a button according to the color of the stimulus and ignore its location. When color and location do not match, they create incongruent (conflict) situations that yield longer reaction time (RT) and decreased accuracy (van den Wildenberg et al., 2010). This effect, called the congruence effect, can be explained by the dual-process hypothesis that states that stimulus processing follows two parallel routes: an automatic one that leads to fast response, and a controlled one that selects the most appropriate action (Kornblum et al., 1990). The activation-suppression model further suggests that, in incongruent situations, automatic responses are selectively inhibited, in order to achieve the correct response, but this process takes time (Ridderinkhof, 2002). The irrelevant dimension of the stimulus induces more capture when responses are fast, resulting in more impulsive errors in incongruent situations. Conversely, the congruence effect (reflected by the difference between RTs for correct responses in incongruent vs. congruent situations) decreases as the time taken to respond increases and the selective inhibition process builds up (Richard Ridderinkhof et al., 2011). Considering imaging and behavioral evidences in favor of an active suppression mechanism being more effective with time (Forstmann et al., 2008; Wylie et al., 2010), the activation-suppression model seems to be a good way to provide insights on the temporal dynamics of cognitive action control. Hence, both the strength of the capture by the irrelevant stimulus dimension and the ability to selectively inhibit unwanted responses are now considered as key components of cognitive action control, and it is therefore necessary to assess both aspects, in order to achieve a more comprehensive view of this process (van den Wildenberg et al., 2010).

Several studies have investigated the impact of age on cognitive action control using conflict tasks, mostly by comparing older than 60 year old adults to younger adults from 20 years to 30 years old. Although some studies did not confirm this effect (Wild-Wall et al., 2008; Salthouse, 2010; Hsieh et al., 2012), the most common finding was that older adults exhibited a stronger congruence effect on RTs than younger adults (West and Alain, 2000; Bialystok et al., 2004; Germain and Collette, 2008; Duchek et al., 2009). This effect was not due to a general slowing (van der Lubbe and Verleger, 2002; Maylor et al., 2011). Evidence for an age-related effect of congruence on accuracy is scarcer. Some studies have shown that older participants displayed a stronger congruence effect on errors (Proctor et al., 2005), but most studies have failed to find such an effect (van der Lubbe and Verleger, 2002; Germain and Collette, 2008; Duchek et al., 2009; Maylor et al., 2011). Furthermore, in the context of age-related effects, none of the above studies have distinguished impulsive selection from impulsive suppression according to the activation-suppression model, distinction which would give insights on the dynamics of cognitive action control.

Indeed, only few studies have investigated the age-related effect on cognitive action control using the activation-suppression model. These studies did not investigate impulsive response selection but focused on the selective inhibition. However, results are again controversial. Some authors have reported the same dynamic pattern of impulse suppression in both younger and older adults, suggesting that age did not alter the ability to selectively suppress unwanted responses (van der Lubbe and Verleger, 2002; Proctor et al., 2005; Kubo-Kawai and Kawai, 2010). Conversely, other authors have reported an increased congruence effect for late responses, which would indicate that older adults had greater difficulties in suppressing impulsive responses (Castel et al., 2007; Juncos-Rabadán et al., 2008). This increased difficulty in older adults is in line with previous studies using stop signal tasks or antisaccades paradigms have shown more difficulties in older adults (Munoz et al., 1998; Bedard et al., 2002; Klein et al., 2005; Olk and Jin, 2011). In antisaccades tasks, the difficulty in inhibiting eye-movements toward a stimulus to gaze into the opposite direction is more pronounced in older adults (for a review see Chen and Machado, 2016). Overall, we lack a comprehensive view regarding the effect of age on the cognitive action control, especially concerning the impulsive response selection.

Few studies have investigated the effect of moderate aging on cognitive action control and its dynamics (Juncos-Rabadán et al., 2008; Wild-Wall et al., 2008). Interestingly, some of these studies have reported a cognitive action control change that was already present in middle-aged participants (van der Lubbe and Verleger, 2002; Salthouse, 2010). This suggests that cognitive action control changes may occur earlier than expected. For instance, Juncos-Rabadán et al. (2008) reported that middle-aged adults (from 50 to 60 years old) experienced impaired selective inhibition. However, to our knowledge, no study has investigated on both impulsive response selection and suppression in middle-aged adults, both components being essential for a comprehensive view of cognitive action control.

In this context, the aim of the current study was to determine the effects of age on the dynamics of cognitive action control in middle-aged subjects according to the activation-suppression model. We used an oculomotor adaptation of the Simon task in order to study both the strength of automatic activation, as assessed with conditional accuracy functions (CAFs), and the ability to selectively inhibit automatic responses, as assessed with delta plots, which show the congruence effect according to the RT distribution of the correct responses (van den Wildenberg et al., 2010). In a previous study, we investigated whether the evaluation of the dynamics of cognitive action was specific to the response modality and proposed this oculomotor version of the Simon task to a group of young and healthy participants (Duprez et al., 2016). We reported results in line with the activation-suppression model showing a strong automatic activation for fast responses and a congruence effect that decreased with time reflecting the gradual build up of selective inhibition.

## Materials and Methods

### Participants

We compared a group of middle-aged patients (*n* = 39, 26 women; mean age ± *SD* = 57.2 ± 9 years; mean education level = 13.4 ± 2.6 years) to a group of young participants (*n* = 43, 27 women; mean age ± *SD* = 23.7 ± 3.5 years; mean education level = 15.4 ± 1.4 years). All participants, recruited by advertising, underwent an extensive interview beforehand to ensure that they had no history of neurological or psychiatric disorders and no recent drug use. All participants had a standard score higher than 8 at the matrix subset of the Weschler Adult Intelligence Scale (WAIS-IV), excluding cognitive alteration (Wechsler, 2008). All participants had normal or corrected-to-normal vision, and gave their informed written consent. The experiment was approved by the local ethics committee of Rennes University Hospital.

### Stimuli and Apparatus

**Figure 1:**
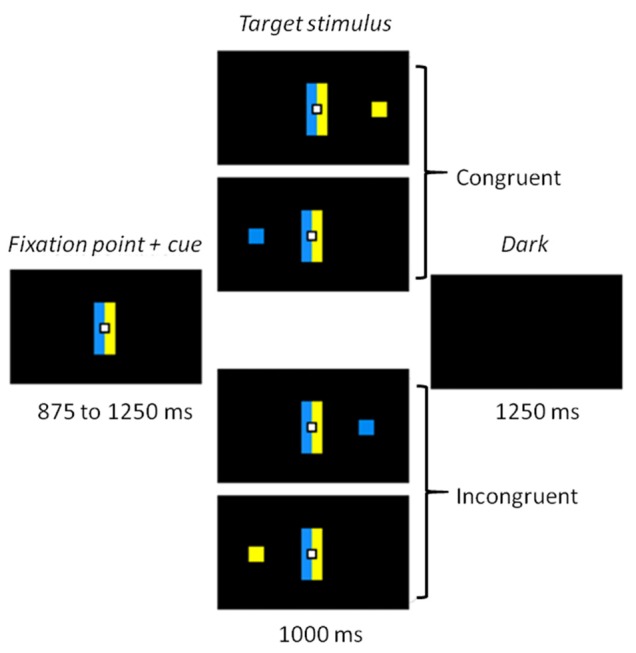
**Experimental task.** Participants had to make a left or right eye movement according to the color of the target stimulus and ignore its location. Rectangle cues flanking the fixation point held the color-response mapping constant. When the side indicated by the color matched the location of the target, the trial was congruent. When color and location did not match, the trial was incongruent.

The whole task was conducted in darkness, to ensure the quality of the eye movement recordings, with dim light interruptions during interblock intervals. Participants sat 60 cm away from a 22-inch screen (screen frequency: 60 Hz). The task was designed using MeyeParadigm^®^ Software (Version 1.18, e(ye)BRAIN)^1^. A central white square (0.6 cm in height and width) was used as a fixation point, flanked by two contiguous rectangular cues (5 cm in height and 1 cm in width), a blue one and a yellow one (Figure 1). The color side of these cues was pseudorandomly reversed across participants. The target of each trial was a blue or yellow square (0.6 cm in height and width) presented at a 12° visual angle either on the left or on the right. The targets were displayed pseudorandomly, with an equal number of 2 (color) × 2 (location) combinations. The task contained 300 trials, and was divided into five 3.5-min blocks, each containing 60 trials. The blocks were separated by short breaks to prevent tiredness. Each trial started with the display of the central fixation point and the contiguous rectangle cues for 875–1250 ms (125-ms pseudorandom steps). The target was then displayed on the left or right side of the screen for 1000 ms. The trial ended with a 1250 ms black screen before the next one started. A 16-trial practice block was administered before the experimental phase, to familiarize participants with the task.

### Procedure

Participants were asked to make a left or right eye movement according to the color of the target as fast and as accurately as possible, and to ignore its location. For half the participants in each group, when the blue cue was on the left, a blue stimulus indicated a left response and a yellow stimulus a right response (Figure 1). The instructions and sides of the cues were reversed for the other half. The cue rectangles remained on the screen when the stimulus appeared, to keep the instructions available. The combination of target location and color induced two conditions: a congruent one, when the stimulus appeared on the side corresponding to the saccade direction dictated by its color; and an incongruent one when it appeared on the opposite side.

### Eye Movement Recordings

The overall procedure in this study was similar to the one we previously reported (Duprez et al., 2016). Eye movements were recorded using an EyeBrain T2^®^ head-mounted eyetracker (e(ye)BRAIN^®^)^2^ at a sampling rate of 300 Hz and an angular resolution of 0.5°. Saccade RTs and directions were analyzed off line with MeyeAnalysis^®^ Software (e(ye)BRAIN^®^), which uses a detection algorithm adapted from Nyström and Holmqvist (2010). We retrospectively proceeded to a manual inspection of each step of the analysis.

### Data Analysis

Despite our task is based on ocular movements, it differs from classical antisaccade and countermanding tasks assessing both saccade inhibition. It in fact relies on conflict induced by a two-dimensional stimulus and allows studying both the dynamics of impulsive selection and suppression (Hanes and Carpenter, 1999). In this context, our analysis was based on the activation-suppression model rather than models on saccade latency distributions and error responses such as the LATER model (Noorani and Carpenter, 2013).

The first eye movement after the stimulus presentation was taken to be the participant’s response. When a participant performed a saccade according to the color of the target and not its location, it was counted as a correct response. We excluded saccades with an amplitude below 2° (to discard any micromovements around the central fixation point), a latency below 100 ms (to discard any anticipated saccades; for a review see Leigh and Zee, 1999) or above 1000 ms (corresponding to the target duration), and outlier latencies more than three standard deviations from the mean RT. With these parameters, 1.07% of the whole dataset were removed from the analyses.

RTs and accuracy were represented using both mean values and a distributional analysis based on the activation-suppression model (Ridderinkhof, 2002). For all the statistical analyses, RTs were log-transformed to avoid the measurement bias due to general slowing, accordingly to previous studies (Kray and Lindenberger, 2000; Mayr, 2001; van der Lubbe and Verleger, 2002; Maylor et al., 2011; Olk and Jin, 2011). Raw RTs were used for the graphical representations. Mean RTs for correct trials and accuracy were calculated for each group and in each congruence condition, to represent the effect of age on the mean congruence effect. CAFs displaying accuracy levels as a function of RT were used to assess automatic response activation elicited by the location of the stimulus. To this end, accuracy was plotted against the RT distribution for congruent and incongruent trials. For each participant, RTs were rank-ordered and split into seven bins (septiles) according to previous studies (Wylie et al., 2010). Each bin contained an equal number of trials (20 trials per bin and per subject, in each condition). The mean accuracy was then plotted for each bin. To visualize the strength of selective inhibition, delta plots were used to show the congruence effect as a function of RT. This effect is reflected by the difference between congruent and incongruent RTs calculated for correct trials only, as it reveals the additional time needed to give a correct response in conflict situations. RTs of correct responses were split into seven bins, and mean delta values were calculated and plotted against the RT distribution for each bin.

### Statistical Analysis

Data management and statistical analyses were performed using R^© ^ Software (Version 3.1.0) with the nlme (Pinheiro et al., 2014 [S version]) and lme4 (Bates et al., 2014) packages. We compared the log RTs between congruence conditions and groups using a linear mixed model, considering two fixed effects of congruence (congruent vs. incongruent) and group (young and middle-aged participants) and a random participant effect. As accuracy is a binary parameter, it was compared using a nonlinear mixed model (with the same fixed and random effects). These models allowed us to work on the whole dataset and avoid the loss of power that comes with averaging data, while taking interindividual variability into account. The CAF analysis was performed according to the same nonlinear mixed model, to which we added a *bin* fixed effect, corresponding to the RT septiles. This resulted in a 2 (congruence) × 2 (group) × 7 (bin) analysis. We then focused on the first bin of the distribution, as this reflects the most automatic response activation (van den Wildenberg et al., 2010). Concerning the delta plots, we compared the strength of selective inhibition between groups. With this aim, we computed and analyzed slope values, which usually serve as a measure of the strength of selective inhibition (Ridderinkhof, 2002). Slope values were compared using a linear mixed model with slope (position in the RT distribution) and group as fixed effects, and participants as a random effect. This resulted in a 2 (group) × 5 (slope) analysis. As selective inhibition is considered to be more effective when RTs are long (Ridderinkhof, 2002), our final analysis focused on the last slope of the delta plots. All *p*-values regarding analyses of variance based on the linear mixed models were computed using the ANOVA function that uses F-tests while *p*-values for non-linear mixed models were computed using the ANOVA function that uses Wald Chi-square tests (ANOVA car). All the models that were fitted yielded an overall *R*^2^ > 0.25 which was calculated by correlating fitted and observed values. Partial *R*^2^ were not calculated since there is no standardized calculation method available for mixed models.

## Results

### RTs and Accuracy

**Figure 2:**
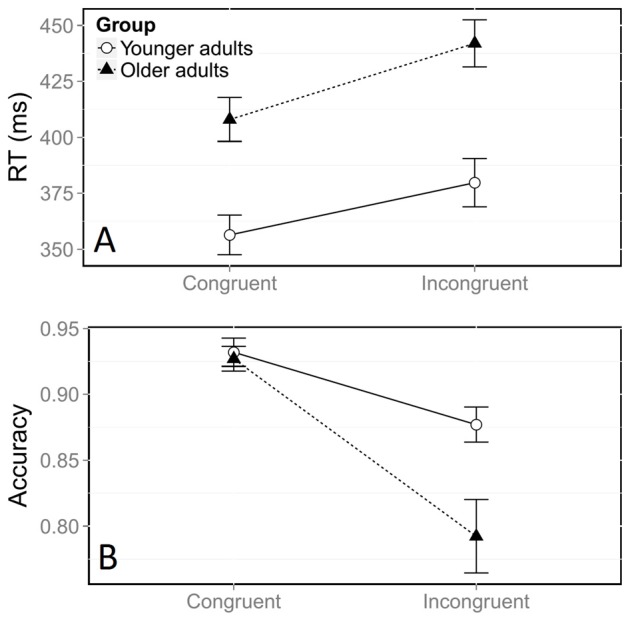
**Mean reaction time (RT, A) and accuracy (B) according to congruence and group.** Error bars represent the standard error of the mean.

There was a large general congruence effect on log RTs, with incongruent trials yielding longer RTs than congruent trials (Figure 2A, *F*_(1,21059)_ = 678.41, *p* < 0.0001). Overall log RTs were longer for middle-aged adults than for young ones, (*F*_(1,38)_ = 18.05, *p* < 0.0001). This corresponded to a difference of 12 ms in the congruence effect measured on raw RTs (35 ms in middle-aged participants vs. 23 ms in young participants). The significant interaction between congruence and group (*F*_(1,21059)_ = 4.65, *p* = 0.03) indicated that the congruence effect on log RTs was stronger for older adults.

The error rate, which was significantly higher in the incongruent situation, also indicated a large effect of congruence on accuracy (Figure 2B, $\chi_{\left(1,23848\right)}^{2}$ = 482.53, *p* < 0.0001). Overall, older adults made more errors than younger adults ($\chi_{\left(1,38\right)}^{2}$ = 5.22, *p* = 0.02). The significant interaction between congruence and group ($\chi_{\left(1,23848\right)}^{2}$ = 45.38, *p* < 0.0001) confirmed that the effect of congruence on accuracy was stronger in older adults. Further analyses confirmed that this stronger congruence effect was due to a higher rate of errors in the incongruent condition, in comparison with younger participants (Figure 2B). Older adults had an accuracy rate of 79% in the incongruent condition, compared with 88% in younger adults ($\chi_{\left(1,38\right)}^{2}$ = 7.45, *p* = 0.006), whereas both groups were equally accurate in the congruent condition ($\chi_{\left(1,38\right)}^{2}$ = 0.56, *p* = 0.45).

### Automatic Activation

**Figure 3:**
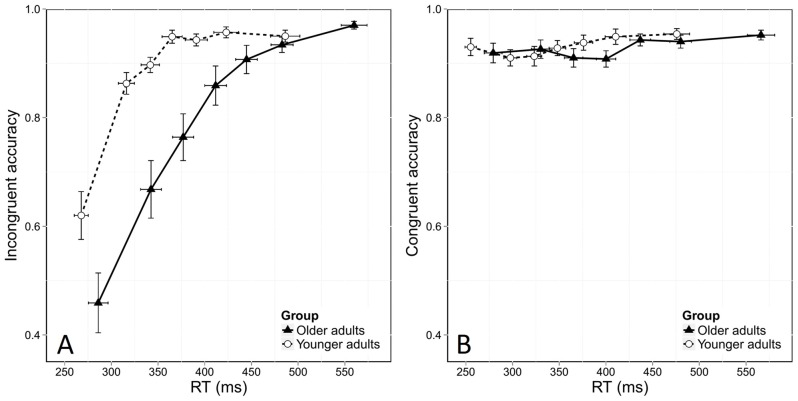
**Conditional accuracy functions (CAFs) for the incongruent (A) and congruent (B) conditions, plotted as a function of RT distribution and age group.** Error bars represent the standard error of the mean.

Figure 3 shows the usual dynamics of accuracy as a function of RT in both the incongruent and congruent conditions. To assess these dynamics, we used the same model as before, but added the bin factor. We found the same overall effects of age and congruence in this analysis, with lower accuracy in the incongruent condition ($\chi_{\left(1,23848\right)}^{2}$ = 375.8, *p* < 0.0001) and in older adults ($\chi_{\left(1,38\right)}^{2}$ = 5.15, *p* = 0.02). The stronger effect of congruence on accuracy in older adults was again confirmed by a significant interaction between group and congruence ($\chi_{\left(1,23848\right)}^{2}$ = 61.29, *p* < 0.0001). This stronger congruence effect was due to the poorer accuracy of older adults in the incongruent condition, as younger and older adults were equally accurate in the congruent situation. This was confirmed by further analyses that did not reveal any effect of age in the congruent condition (Figure 3B, $\chi_{\left(1,38\right)}^{2}$ = 0.56, *p* = 0.45) but a significant effect of age in the incongruent condition ($\chi_{\left(1,38\right)}^{2}$ = 7.37, *p* < 0.0001).

In addition, analyses revealed that accuracy depended on RT, as shown by a significant bin effect ($\chi_{\left(6,23848\right)}^{2}$ = 1109.44, *p* < 0.0001). Figure 3A shows that more errors were made in the incongruent condition when responses were fast. This was confirmed by a significant interaction effect between congruence and bin ($\chi_{\left(1,6\right)}^{2}$ = 27.46, *p* < 0.0001). Response dynamics were also influenced by age, as revealed by a significant interaction between bin and group ($\chi_{\left(6,38\right)}^{2}$ = 27.46, *p* < 0.0001). Finally, the temporal dynamics of accuracy was also dependent on both group and congruence, as there was a significant Congruence *Group *Bin interaction ($\chi_{\left(6,23848\right)}^{2}$ = 29.59, *p* < 0.0001).

Finally, specific analyses of the first bin of the distribution, which is considered to be the most informative on the strength of automatic activation, revealed that older adults made more fast errors than younger ones, with an accuracy rate of 46% in older participants vs. 62% in younger ones ($\chi_{\left(1,38\right)}^{2}$ = 5.56, *p* = 0.01). This means that older adults displayed stronger automatic activation than younger participants in incongruent conditions.

### Impulse Suppression

**Figure 4:**
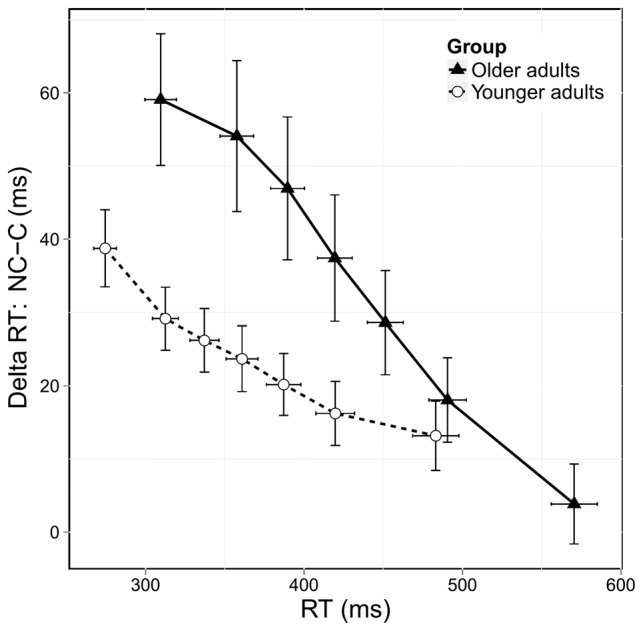
**Delta plots showing delta RT (incongruent [NC] RTs—congruent [C] RTs) according to RT distribution and age group.** Error bars represent the standard error of the mean.

Figure 4 shows the delta plots displaying the congruence effect, calculated on correct responses, as a function of RT. In both groups, the congruence effect decreased across RTs. However, older adults exhibited a steeper reduction in this effect than younger adults. Analysis of the overall slope of the congruence effect showed a significant group effect (*F*_(1,38)_ = 3.83, *p* = 0.05) confirming that the reduction in the congruence effect was stronger in older adults. We then investigated the effect of age on the slope of the final segment of the delta plots, which is considered to be the most informative regarding the efficiency of selective inhibition. As shown in Figure 4, older adults had a steeper downward slope in this final segment, and the analysis revealed a trend toward significance (*F*_(1,38)_ = 2.98, *p* = 0.08). Despite only approaching significance, this result nonetheless suggests that older adults displayed stronger selective inhibition than younger ones.

## Discussion

The purpose of this study was to assess the effects of age on cognitive action control in middle-aged subjects as some studies have suggested an early impairment in this process. We sought to test whether the dynamics of automatic activation and the selective suppression of unwanted responses evolve from younger to middle-aged adults. To this end, we administered an adaptation of the Simon task to young and middle-aged healthy participants, using eye movement recordings, and interpreted the results within the framework of the activation-suppression model (Ridderinkhof, 2002).

We first showed that resolving conflict required greater effort in middle-aged participants. The effect of congruence on accurate RTs was greater in the older group, in accordance with the literature (Pick and Proctor, 1999; Maylor et al., 2011; Aisenberg et al., 2014). For instance, van der Lubbe and Verleger (2002) administered a Simon task in which participants had to press buttons according to the letter that was displayed either on the left or the right side of the screen, and ignore its location. They found that the congruence effect was greater in older participants than in younger ones. As in our study, this effect persisted after RTs had been log-transformed to correct for general slowing, indicating that it was specifically due to an age-related change in cognitive action control (Kray and Lindenberger, 2000; Mayr, 2001; van der Lubbe and Verleger, 2002; Maylor et al., 2011; Olk and Jin, 2011). These results confirm that in older adults, independently of any general slowing, greater efforts are needed to resolve conflict triggered by irrelevant information about stimulus location.

Second, we found a stronger congruence effect in middle-aged participants when we analyzed accuracy, as older adults made more errors than younger adults in the incongruent condition. This confirms that, in addition to the greater cost of conflict in terms of RTs, older participants have greater difficulties performing the correct action when there is conflict between automatic and controlled responses. Contrary to the effect of age on RTs, the effect on accuracy has only rarely been observed, probably owing to methodological limitations to show this effect. Most of the studies that found an impact of age on the congruence effect for RTs but not for accuracy, used a Simon task with arrows or letters to generate conflict situations (van der Lubbe and Verleger, 2002; Castel et al., 2007; Germain and Collette, 2008; Juncos-Rabadán et al., 2008; Duchek et al., 2009; Tse et al., 2010; Maylor et al., 2011; Sebastian et al., 2013). An effect on accuracy was, however, found when using color stimuli (Bialystok et al., 2004; Proctor et al., 2005). This is in line with studies demonstrating that the nature of the stimuli is of critical importance, as it has a significant impact on the strength of the conflict (Wascher et al., 1999; Mattler, 2003). Our results are also in line with the detrimental effect of age on inhibition processes revealed by antisaccade tasks (for a review see Chen and Machado, 2016).

Thus, this study supports the rare data suggesting that changes in cognitive action control occur between young and middle-aged adults. In order to better assess the effects of age on the strength of automatic activation and the ability to selectively inhibit automatic responses, we further analyzed our results using distributional analyses. This method, which is considered to be highly effective in describing the dynamics of cognitive action control, uses CAFs and delta plots to display accuracy and the congruence effect as a function of RT, based on the activation-suppression model (Ridderinkhof, 2002). According to this model and in the Simon task, the strength of automatic activation is best illustrated by the first bin of the CAF, as the rate of fast errors reveals the strength of automatic activation (van den Wildenberg et al., 2010). Further, the ability to selectively inhibit unwanted responses appears in the decrease in the congruence effect over time. This selective inhibition is most effective with longer RTs in the Simon task. Also, a steeper decrease indicates stronger selective inhibition and the slope of the last segment of the delta plots is considered to be the most informative, regarding the strength of selective inhibition (Ridderinkhof, 2002). Although some authors have studied the effect of age on inhibition using delta plots, to our knowledge none has concomitantly studied the capture of impulsive responses, which would give a more comprehensive view of the cognitive action control.

According to the activation-suppression model, we observed the typical CAF pattern, with most errors corresponding to short RTs (i.e., fast responses) in the incongruent condition, and a very high accuracy in the congruent condition, whatever the RT. Comparison of groups showed that middle-aged adults exhibited a higher rate of fast errors (revealed in the first CAF bin) in the incongruent condition. This means that the automatic activation of the response induced by the irrelevant location of the stimulus was stronger in older participants. In other words, faster reactions in older adults were more vulnerable to impulsive actions than in younger adults. This result has never been described using a conflict task such as the Simon Task but is in line with the effect of age observed using an oculomotor capture task (Ridderinkhof and Wijnen, 2011). In this study, participants had to look at a target while ignoring the simultaneous appearance of a visual distractor. Analysis of the first CAF bin revealed that older adults (over 60 years old) made more saccades toward the distractor than younger adults. The authors interpreted this finding as an increase in oculomotor capture in older participants.

Imaging studies of the neural substrates of automatic activation can help understanding the above results. Forstmann et al. (2008) used a combination of distributional analyses and functional magnetic resonance imaging (fMRI) to investigate the neural substrates of activation and suppression during a Simon task. They found that stronger automatic activation, reflected by fast responses in incongruent conditions, was linked to greater activation in the pre-supplementary motor area (pre-SMA). The involvement of the pre-SMA in automatic response capture is supported by another study, which reported that the rate of fast errors decreased when the supplementary motor cortex, which includes the pre-SMA, was hyperpolarized, using transcranial direct-current stimulation (Spieser et al., 2015). Furthermore, activity in this area was found to be greater in older participants than in younger adults during a Go-No Go task (Hester et al., 2004; Turner and Spreng, 2012). Nielson et al. (2002) confirmed these results using fMRI in an inhibitory control task that consisted in pressing a button when *X* or *Y* appeared in a stream of other letters, but only when the target letters alternated (e.g., X, Y, X, Y). Responses had to be inhibited when target letters were repeated (e.g., X, X). They showed that the pre-SMA was more active in older participants who poorly inhibited their actions. Taken together, these results suggest that the pre-SMA is overactivated in older participants when they encounter conflict situations, and that greater activation in this region is associated with stronger automatic activation of the response during conflict or inhibitory tasks. This would explain why the older group in our study was found to be more vulnerable to impulsive responses.

Still according to the activation-suppression model, delta plots analysis revealed the well-known pattern of decreasing congruence effect across RTs, meaning that slower reactions were associated with easier conflict resolution. However, changes in the congruence effect differed between groups, as middle-aged adults displayed a greater decrease than younger adults. The overall downward slope of the congruence effect was significantly steeper in older participants and the last slope tended to differ statistically between older and younger participants. This may be only due to a lack of statistical power. These results suggest that older adults may have displayed greater selective inhibition than younger adults. Results from the literature regarding the effect of age on selective inhibition are inconsistent. Some studies found that older adults were generally more affected by conflict, but displayed the same congruence effect dynamics than younger adults (van der Lubbe and Verleger, 2002; Proctor et al., 2005; Kubo-Kawai and Kawai, 2010). Other studies showed that younger participants exhibited a decreasing congruence effect, whereas older ones exhibited an increasing one (Castel et al., 2007; Juncos-Rabadán et al., 2008). The reasons for such differences are not clear but, as suggested above, the nature of the stimuli may play a role. It is also important to remind that the pattern of evolution of the congruence effect is not homogeneous among the different conflict tasks and that some results might be specific to the experimental design. For instance, the Stroop task (Stroop, 1935) typically yields an increasing pattern of the congruence effect (Pratte et al., 2010). Alternatively, the decreasing pattern of the congruence effect is regularly observed in Simon tasks that use horizontal left and right stimuli (Proctor et al., 2011). However, there is now strong evidence that, at least in the Simon task, the strength of this decrease reflects the efficiency of selective inhibition (Burle et al., 2002; Forstmann et al., 2008; Wylie et al., 2010).

The eventuality of a highest selective inhibition in middle-aged participants could be explained by recent neuroimaging data and theories of compensation exerted by older adults. Forstmann et al. (2008) showed that the strength of selective suppression, indicated by the slope of the last delta plots segment, was significantly associated with greater activity in the inferior frontal cortex (IFC). Several studies have also linked suppression of unwanted action to activity in the IFC (for a review see Aron et al., 2004). Other studies on cognitive aging using fMRI have reported that older participants displayed greater activity in this area in cognitive control tasks such as the Stroop or Simon tasks (Milham et al., 2002; Turner and Spreng, 2012; Sebastian et al., 2013). The explanation seems to lie in theories of additional neural recruitment that compensates for age-related decline (Park and Reuter-Lorenz, 2009). Accordingly, the increase in prefrontal activation in older adults seems to be an adaptive mechanism that helps to compensate for the decline in cognitive functions. Thus, it is possible that, in older adults, the abnormally high sensitivity to irrelevant information could be compensated for by an increased engagement of top-down control. However, this explanation is not in line with the fact that older adults usually show a decrease in the anterior cingulate cortex activity and a decrease of the electrophysiological error-related-negativity, which are both thought to reflect conflict detection/adaptation (Milham et al., 2002; Pardo et al., 2007; Hoffmann and Falkenstein, 2011). As a whole, we can only speculate on the mechanisms that subtend the age-related changes that we observed in delta plots. In addition, since these variations could be task-specific, a firm conclusion on the underlying mechanisms needs further investigations.

Nonetheless, several points need to be kept in mind when interpreting our results. First, we chose to investigate the cognitive action control using a modified Simon Task and the activation-suppression model (Ridderinkhof, 2002). This model, coupled with the Simon Task, has proven to be efficient in detecting interindividual changes and in describing the temporal course of response activation and suppression (Burle et al., 2002; Ridderinkhof, 2002; Forstmann et al., 2008; Wylie et al., 2010; Spieser et al., 2015). However, this model does not yet fully account for some results from other conflict tasks. It should also be highlighted that middle-aged adults had a higher congruence effect than younger adults, which was evident at the very beginning of the delta plots distribution. This initially higher congruence effect was also observed in previous studies (Proctor et al., 2005; Castel et al., 2007; Juncos-Rabadán et al., 2008; Kubo-Kawai and Kawai, 2010). We may wonder about the meaning of this initially higher congruence effect. The activation-suppression model postulates that the strength in selective inhibition is portrayed by the steepness of the decrease of the congruence effect, justifying the special focus on the slopes of the delta plots. Accordingly, a sharp decrease would still mean stronger inhibition despite a high initial congruence effect. Furthermore, the older adults’ delta plots reached longer RTs than younger ones and the congruence effect for those RTs became lower than for younger adults. Altogether, these observations raise the question of different responding strategies in terms of speed-accuracy trade-offs in the older group (Smith and Brewer, 1995). In this perspective, older adults in our study have responded more slowly than younger adults, which could have allowed selective inhibition to be more efficient, compensating in turn for their difficulties in exerting a fast efficient inhibition.

## Conclusion

Altogether, the present study argues for age-related changes in the dynamics of the cognitive action control. Our results showed that middle-aged adults have difficulty with cognitive action control, by revealing a stronger effect of congruence on both RTs and accuracy. Older adults in our study had difficulty providing intention-driven responses in a conflict situation, and when they did, the cost of resolving this conflict was higher than for younger adults. Investigation of automatic activation and response suppression using distributional analyses showed that older adults were more prone to impulsive fast errors in conflict situations. According to the activation-suppression model, this means that middle-aged adults were more captured than young adults by the irrelevant dimension of the stimulus. Despite there was only a trend regarding the last slope of the delta plots, our results suggest that middle-aged participants may exhibit greater selective inhibition than young adults. This possibility is in line with recent theories suggesting that aging is associated with compensatory mechanisms. A broaden conclusion on the mechanisms subtending our results remains speculative. Further studies using the activation-suppression model in relation to age could provide insights on the interaction between impulsive response selection and inhibition. Such studies could help both the understanding of cognitive aging and the comprehension of cognitive action control mechanisms. In particular, if compensatory mechanisms are involved in middle-aged adults, one could wonder at what age they set up and to what extent they are effective. We may speculate for an inverted-U-shaped evolution of selective inhibition. The process would be stronger in middle-aged adults, reflecting a compensatory mechanism, and would weaken in older ones as the cognitive decline progresses.

## Author Contributions

JD: study conception, data acquisition, data analysis, data interpretation, drafting, final approval. J-FH, FN, TD and PS: study conception, data interpretation, draft revision, final approval. SA and MA: data acquisition, draft revision, final approval. GR, DD and MV: data interpretation, draft revision, final approval.

## Conflict of Interest Statement

The authors declare that the research was conducted in the absence of any commercial or financial relationships that could be construed as a potential conflict of interest.

## Footnotes

^1^www.eye-brain.com

^2^www.eye-brain.com
